# Author Correction: Hot carrier cooling mechanisms in halide perovskites

**DOI:** 10.1038/s41467-017-02301-w

**Published:** 2018-01-11

**Authors:** Jianhui Fu, Qiang Xu, Guifang Han, Bo Wu, Cheng Hon Alfred Huan, Meng Lee Leek, Tze Chien Sum

**Affiliations:** 1 Division of Physics and Applied Physics, School of Physical and Mathematical Sciences, Nanyang Technological University, 21 Nanyang Link, Singapore, 637371 Singapore; 2 Energy Research Institute @NTU (ERI@N), Research Techno Plaza, X-Frontier Block, Level 5, 50 Nanyang Drive, Singapore, 637553 Singapore; 3 Institute of Materials Research and Engineering (IMRE), A*STAR, 2 Fusionopolis Way, Innovis, #08-03 138634 Singapore

Correction to: *Nature Communications* 10.1038/s41467-017-01360-3, Article published online 3 November 2017

The original version of this Article omitted an affiliation of Cheng Hon Alfred Huan: ‘Institute of Materials Research and Engineering (IMRE), A*STAR, 2 Fusionopolis Way, Innovis #08-03 138634, Singapore’.

This has now been corrected in both the PDF and HTML versions of the article.

