# Supplementary material for: Hot carrier cooling mechanisms in halide perovskites
Source: Nat Commun. 2017 Nov 3;8:1300. doi: 10.1038/s41467-017-01360-3 (PMC5670184; doi:10.1038/s41467-017-01360-3)
Supplement: Supplementary file 1 — Supplementary Information [file 41467_2017_1360_MOESM1_ESM.pdf]

Supplementary Figures

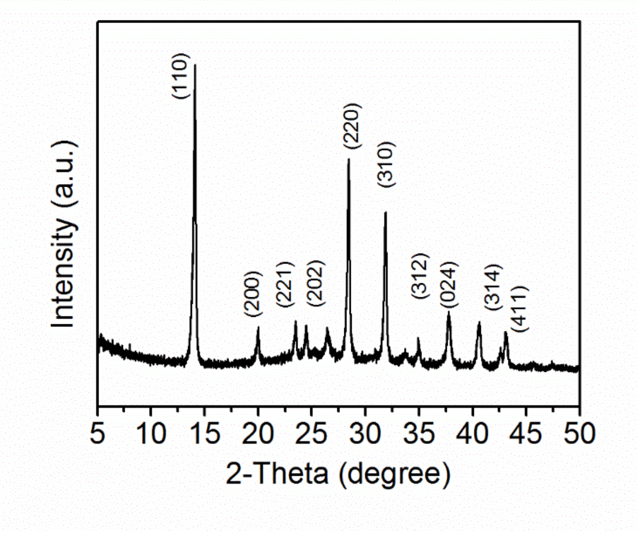

Supplementary Figure 1 XRD pattern of $MAPbI_3$ films on quartz substrates showing the good crystallinity of our samples.

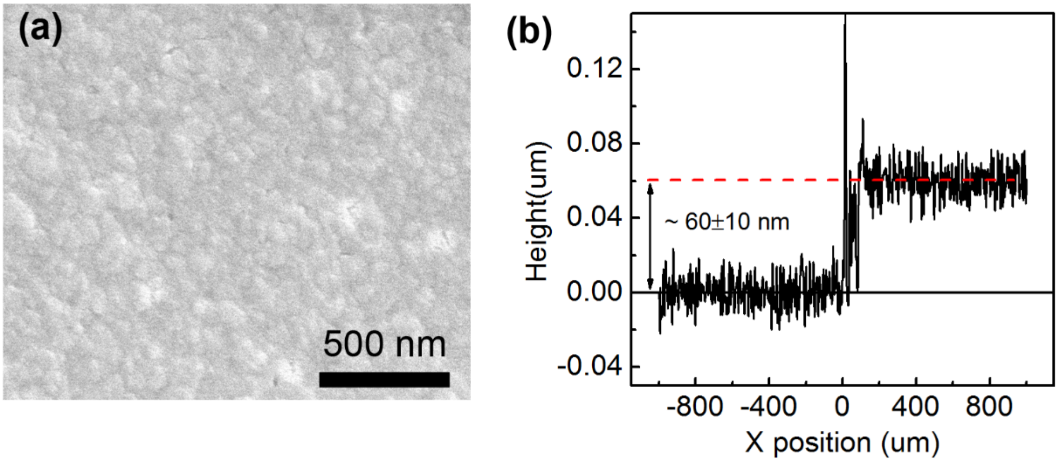

Supplementary Figure 2 (a) SEM surface image of $MAPbI_3$ films on quartz substrates. The grain size of the films is 80±10 nm, indicating the smoothness of the prepared films. **(b)** Step profile of the prepared films. The measured thickness of the films is 60±10 nm.

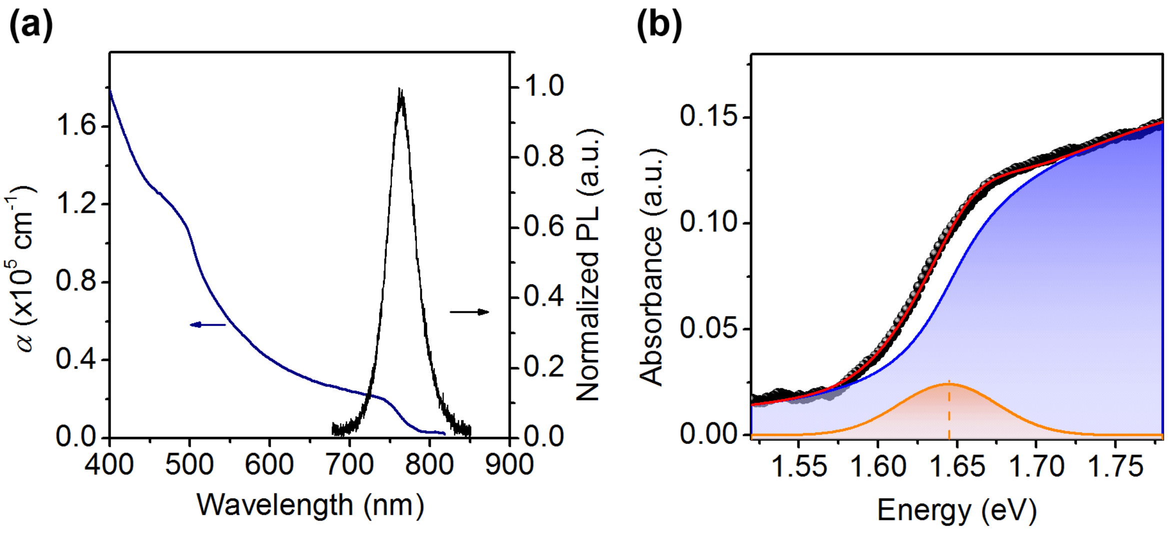

Supplementary Figure 3 (a) Visible linear absorption spectrum and PL spectrum of $MAPbI_3$ films on quartz substrates excited at 2.07 eV with pump fluence of 3.7 μJ cm^{-2}. The absorption spectrum shows two shoulders at around 760 nm and around 480 nm, consistent with earlier reports. **(b)** Absorption spectrum near the band edge and the fitting results based on Elliot's absorption formula. Black sphere: measured absorption spectrum. Orange shading: modeled contribution from discrete exciton transitions. Blue shading: modeled continuum contribution from free carrier transitions. Red line: sum of discrete exciton and free carrier continuum transitions. See Supplementary Note 1.

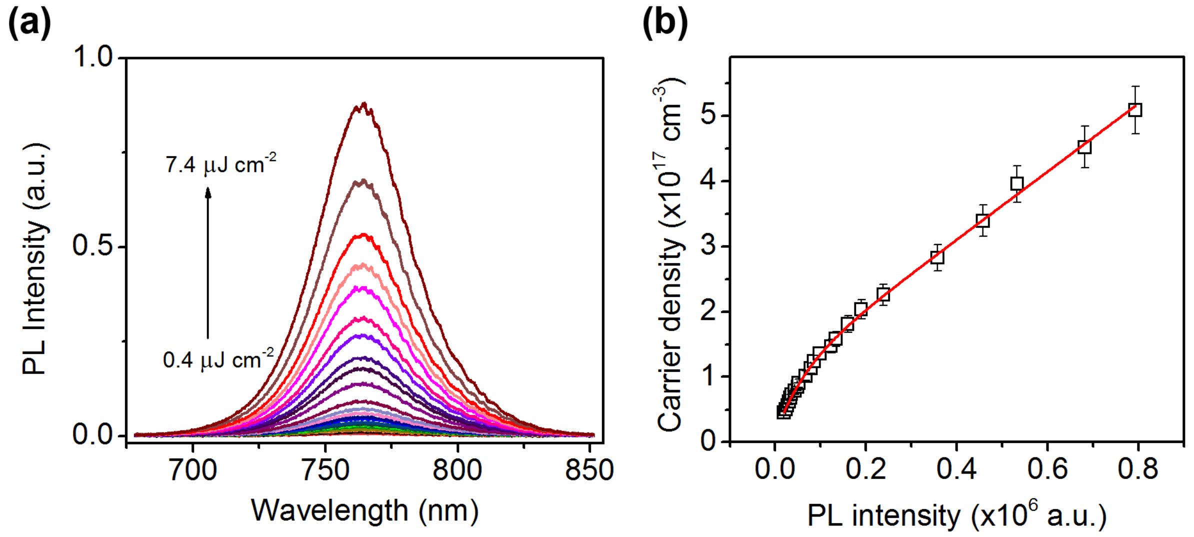

Supplementary Figure 4 (a) Pump fluence dependent time integrated PL intensity of the $MAPbI_3$ films, which was excited at 600 nm with fluence from 0.4 μJ cm^{-2} to 7.4 μJ cm^{-2}. **(b)** Carrier density dependent time integrated PL intensity. Red line: fitting using the trap density model. See Supplementary Note 2.

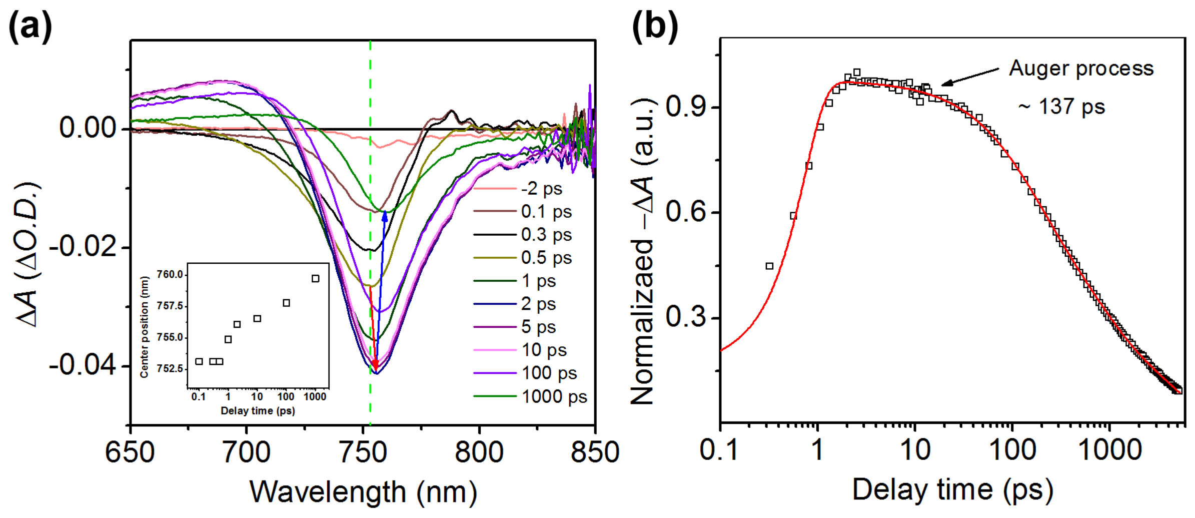

Supplementary Figure 5 (a) Transient absorption spectra of perovskite films with different probe delay times. The spectrum was collected at pump energy of 2.48 eV with carrier density n_0 of 10.4×10^{18} cm^{-3}. Green dashed line serves as vertical guide line for the eye. Red line: fast red-shift of TA valley. Blue: slow red-shift of TA valley. Inset shows the red-shift of the center of the TA valley with delay time **(b)** Normalized band-edge photobleaching kinetics, square dots: experiment data, red line: fitting with third-order exponential decay. The fitted Auger lifetime is around 137 ps which is quantitatively consistent with the estimated value from $\tau_{\text{Auger}} = 1/k_3 n^2 \approx 119\ ps$.

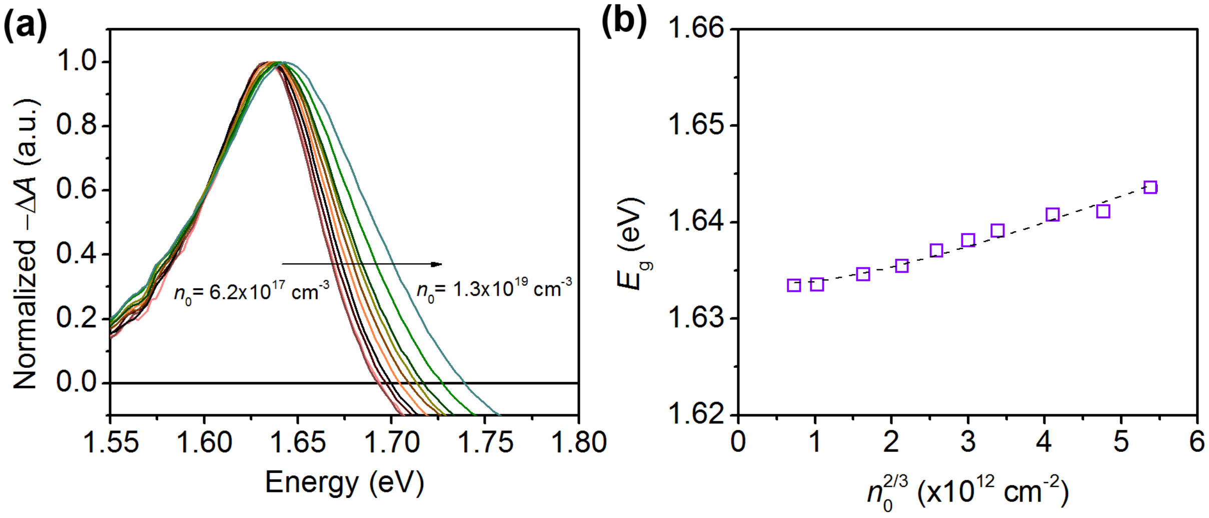

Supplementary Figure 6 (a) Normalized transient absorption spectra of the band-edge transition in $CH_3NH_3PbI_3$ with pump energy of 2.48 eV at variable carrier densities. The spectra were taken at a delay of 2 ps. **(b)** Carrier density dependent bandgap of $CH_3NH_3PbI_3$ derived from **(a)**. Dashed line: A fit of the experimental data with our model (See Supplementary Note 3). Note that the TA data were obtained from our Helios setup with a 750 nm short pass filter placed in the probe path before the sample[1]. The absence of a 750 nm short pass filter to eliminate the residual 800 nm in the white light probe will severely influence the results obtained (See Supplementary Figure 19). Therefore, due care must be taken to ensure that such filter is used when collecting the TA spectra.

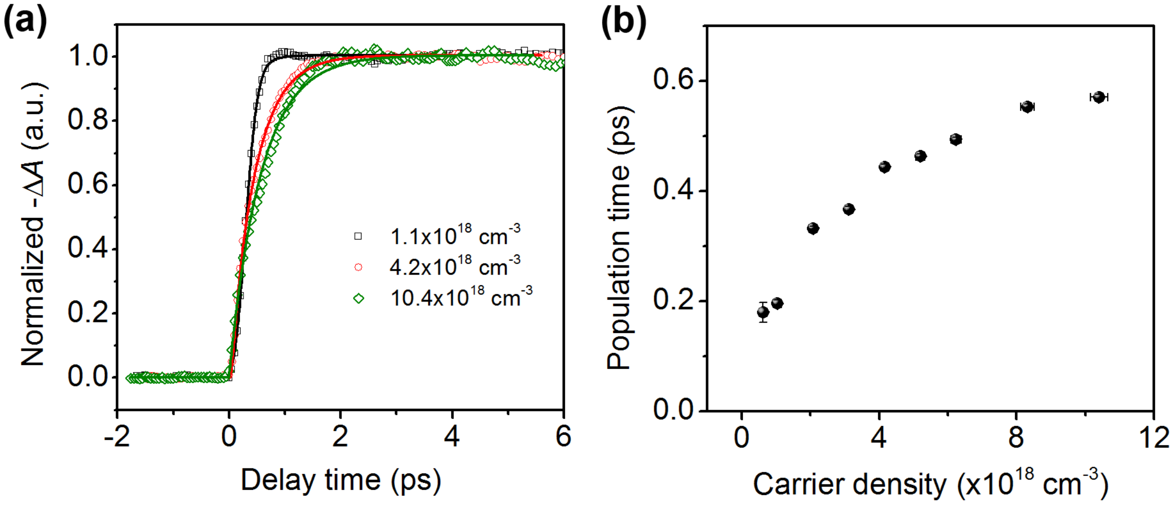

Supplementary Figure 7 **(a)** Normalized band-edge photobleaching kinetics of $CH_3NH_3PbI_3$ films early time after photoexcitation with fixed pump energy of 2.48 eV at variable carrier densities. The rise time is fitted by deconvolving the laser pulse with a Gaussian profile. **(b)** Fitted rise time of band edge TA kinetics for different carrier density.

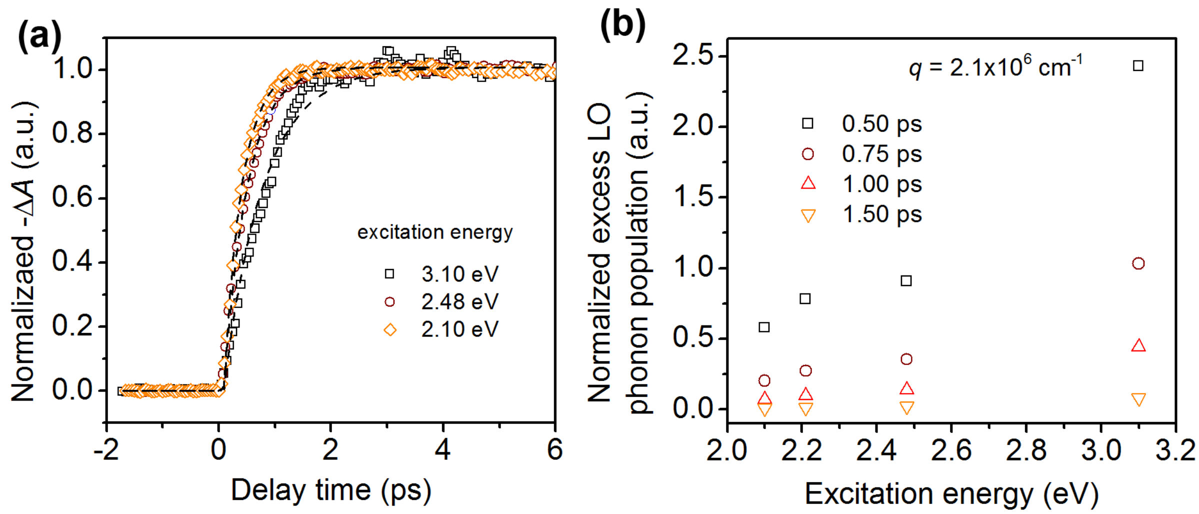

Supplementary Figure 8 (a) Normalized band edge photobleaching kinetics of $CH_3NH_3PbI_3$ films at early times with variable pump energy at fixed carrier density n_0 of 4.2×10^{18} cm^{-3}. The rise time was fitted by deconvolving a laser pulse with Gaussian profile. The band edge TA rise time for excitation energy 2.1 eV, 2.48 eV and 3.1 eV are 0.33 ps, 0.42 ps, 0.70 ps, respectively **(b)** Calculated normalized excess LO phonon population of at variable excitation energy for different delays at q of 2.1×10^6 cm^{-1} (see main text).

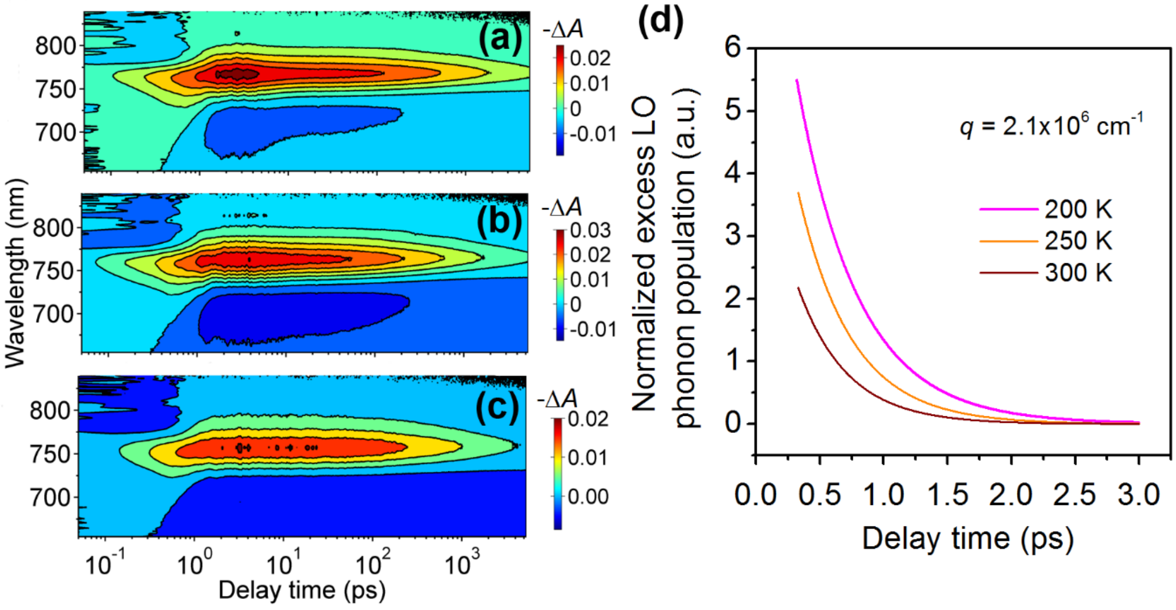

Supplementary Figure 9 Temperature dependent HC cooling dynamics**. (a)**, **(b)** and **(c)** are TA spectra of $MAPbI_3$ films photoexcited at 2.48 eV with an initial carrier density n_0 of 5.5×10^{18} cm^{-3} at 200 K, 250 K and 300 K, respectively. **(d)** Evolution of the calculated normalized excess LO phonon population at different temperatures with q of 2.1×10^{6} cm^{-1}.

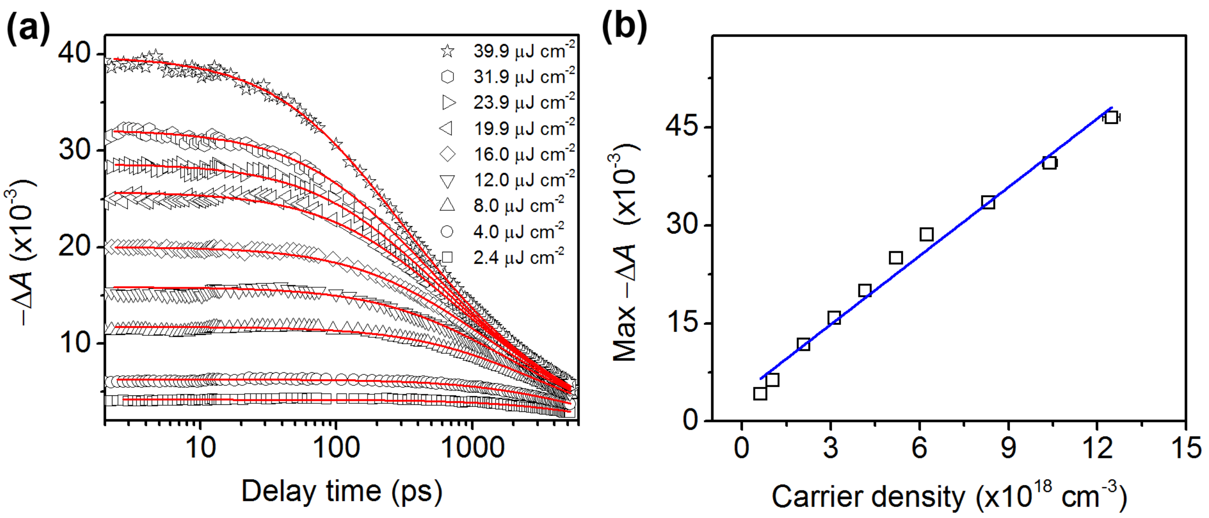

Supplementary Figure 10 **(a)** Band-edge photobleaching kinetics with different fluence and **(b)** carrier density dependent photobleaching amplitudes at a delay of 2 ps. Red lines in **(a)** are the global fits using our model. Blue line in **(b)** is a linear fit. See Supplementary Note 5.

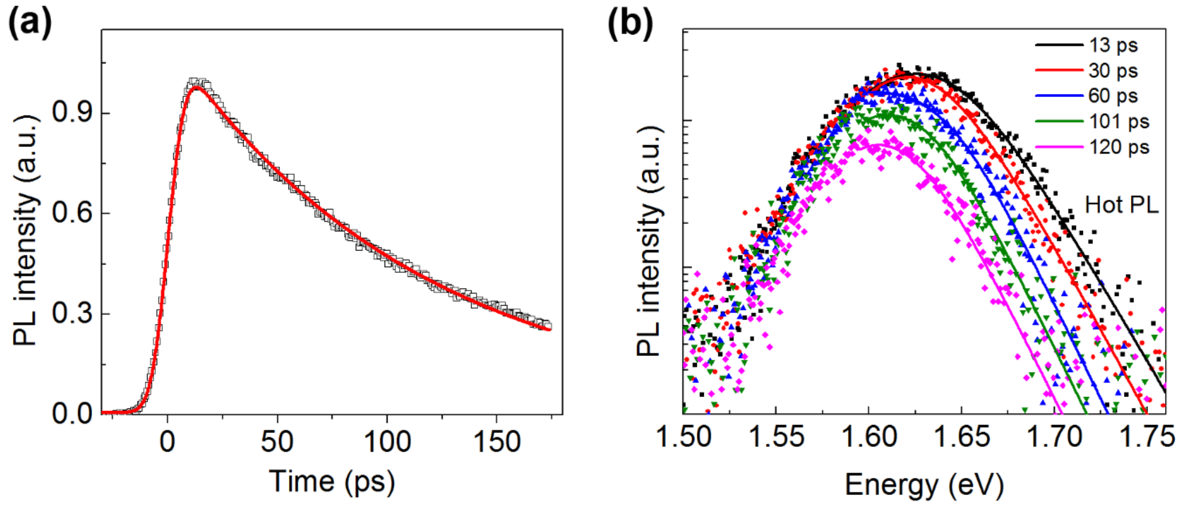

Supplementary Figure 11 (a) TRPL dynamics of $MAPbI_3$ films excited at 2.48 eV with carrier concentration of ~ 3×10^{19} cm^{-3}, the solid line is a single exponential decay with fitted lifetime of around 115 ps. (b) Evolution of the hot photoluminescence spectra at representative delay times longer than 10 ps when Auger heating occurs (streak camera data). The narrowing of the high energy tail with longer delay times indicates that the hot carrier cooling is further slowed down by the Auger heating effect. The solid lines were fitted using: $I(E)\propto\left[g(E)f(E)\right]^2$, where $g(E)=\frac{1}{2\pi^2}\frac{(2m^*)^{3/2}}{h^3}\sqrt{E-E_g}$ is the joint density of states for the symmetric and parabolic bands, $f(E)$ is the Fermi-Dirac distribution function. Together with the observations of rapid shortening of the band-edge PB kinetics (Supplementary Figure 5), multi-particle Auger recombination process is present.

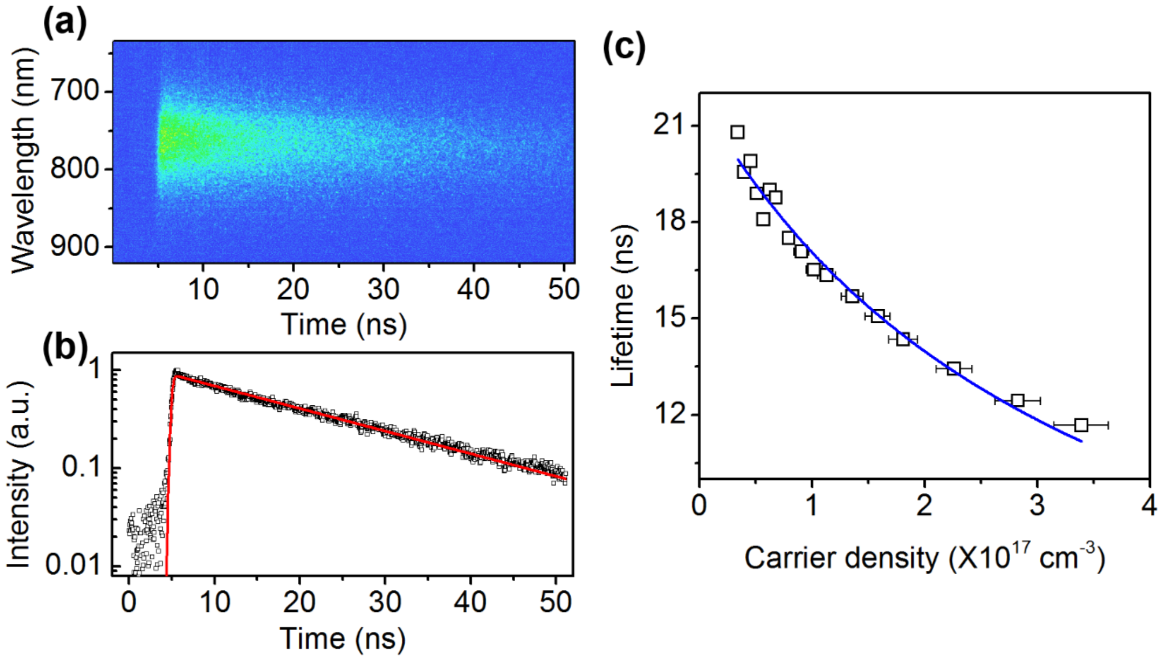

Supplementary Figure 12 (a) Pseudo-color TRPL plot and **(b)** typical TRPL kinetics of $MAPbI_3$ films excited at 2.07 eV with a fluence of 0.7 μJ cm^{-2}, the red line is the mono-exponential decay fitting. **(c)** Carrier density dependent effective PL lifetimes. Blue line: fitting of PL lifetimes using our model below. See Supplementary Note 5.

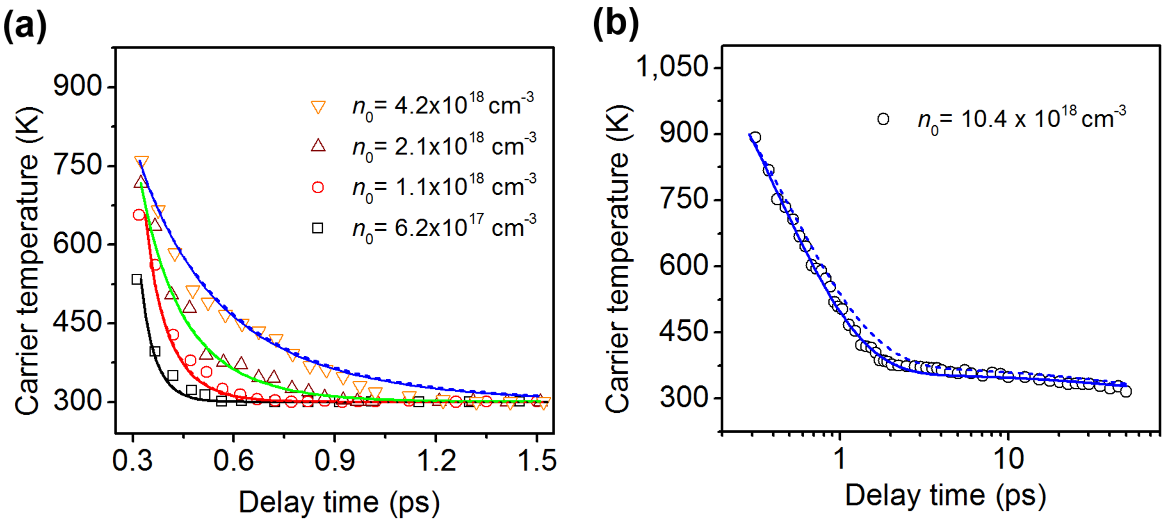

Supplementary Figure 13 Comparison of bare and screened electron-LO phonon coupling on hot carrier cooling dynamics from **(a)** moderate to **(b)** high carrier concentrations. Scatter points: experimental data, solid line: global fitting of bare hot carrier cooling with our model, dashed line: global fitting of screened hot carrier cooling with our model (See Supplementary Note 6). Similarly, we also observed pump fluence dependent slow hot carrier cooling in $CsPbI_3$ films, which is also due to the hot phonon effect instead of the screening effect (Supplementary Figure 18).

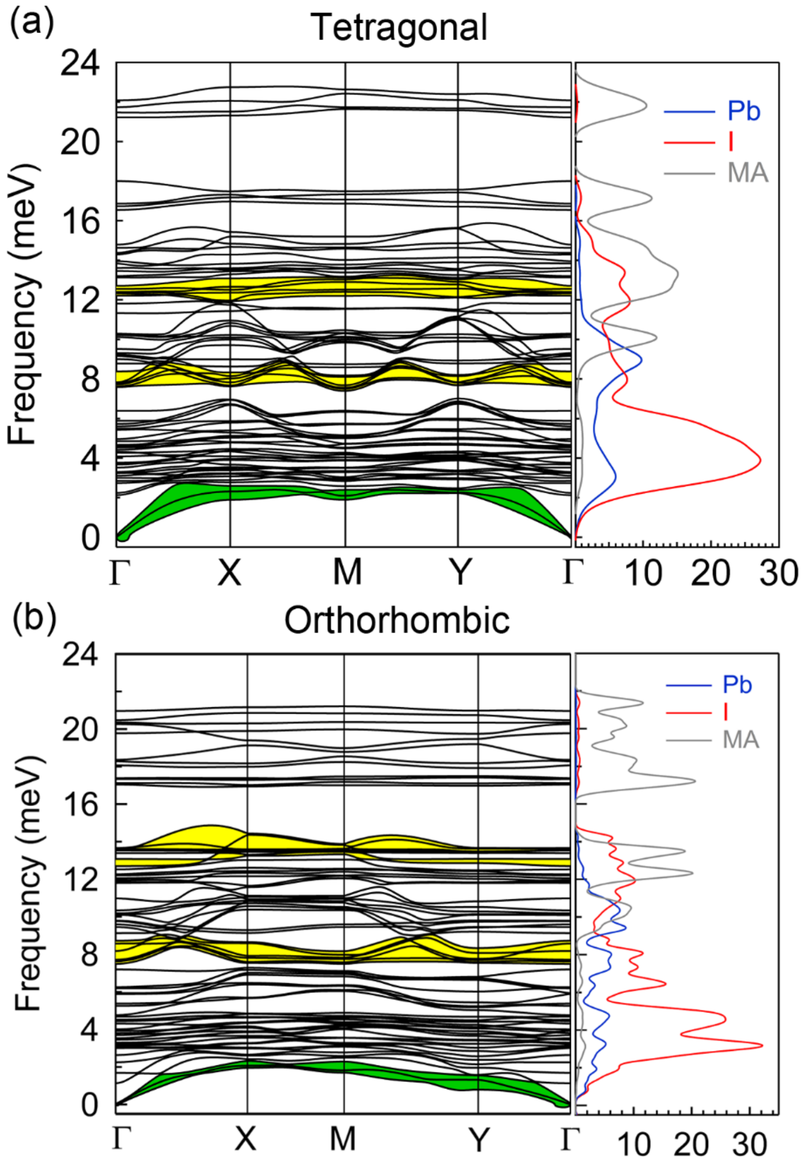

Supplementary Figure 14 Calculated phonon dispersion spectra and the corresponding projected density of states of the $MAPbI_3$ in **(a)** tetragonal and **(b)** orthorhombic phases. LO phonons: yellow zones. Acoustic phonons: green zone. Representative LO phonon modes are shown in Supplementary Figure 15. It is obvious that a large energy separation between LO phonon and LA phonon occurs in $MAPbI_3$ for both Tetragonal and Orthorhombic phases, suggesting that the suppression of Klemens channel persists even at low temperature as well. This also suggests that slow hot carrier cooling of $MAPbI_3$ also exists at the low temperature Orthorhombic phase. See Supplementary Note 9.

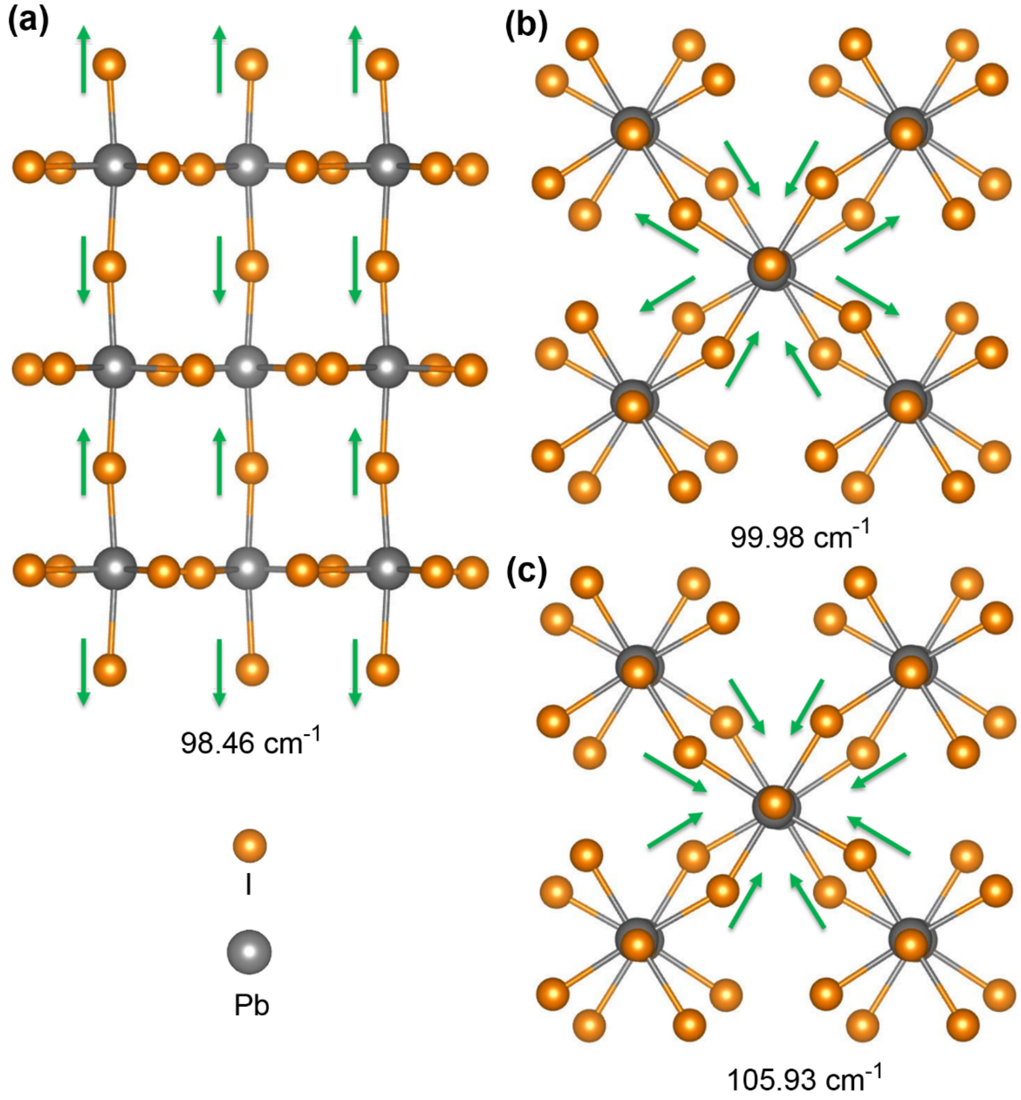

Supplementary Figure 15 Schematic representative LO phonon mode vibration around 100 cm^{-1} of PbI_3 network of tetragonal $MAPbI_3$ at room temperature (MA cations are not shown for clarity). **(a)**, **(b)** and **(c)** are three different types of stretching vibration of lead and iodide atoms. See Supplementary Note 9.

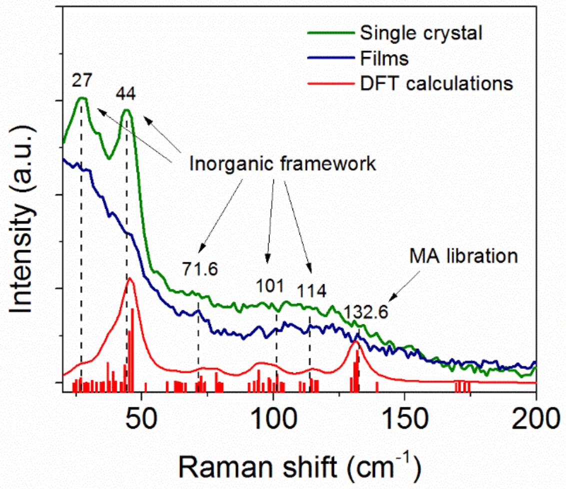

Supplementary Figure 16 Comparison of experimental measurements with DFT calculations. Experimental measurements of Raman spectra for tetragonal $MAPbI_3$ films (green line) and single crystal (blue line) with an excitation wavelength of 532 nm at 170 K. The LO phonon modes of Pb^{2+} and I^- vibration lies at around 71.6 cm^{-1} (around 9 meV), 101 cm^{-1} (around 12.5 meV) and 114 cm^{-1} (around 14 meV), in agreement with the recent reports[2-4]. The other two peaks centered around 27 cm^{-1} and 44 cm^{-1} correspond to the TO phonon modes. The measured LO phonon position corresponds to the upper yellow zone in Supplementary Figure 14 and representative LO phonon modes are shown in Supplementary Figure 15. The theoretical Raman spectra were calculated by convolving the phonon modes with Lorentzian function with a FWHM of 8 cm^{-1}. See Supplementary Note 9.

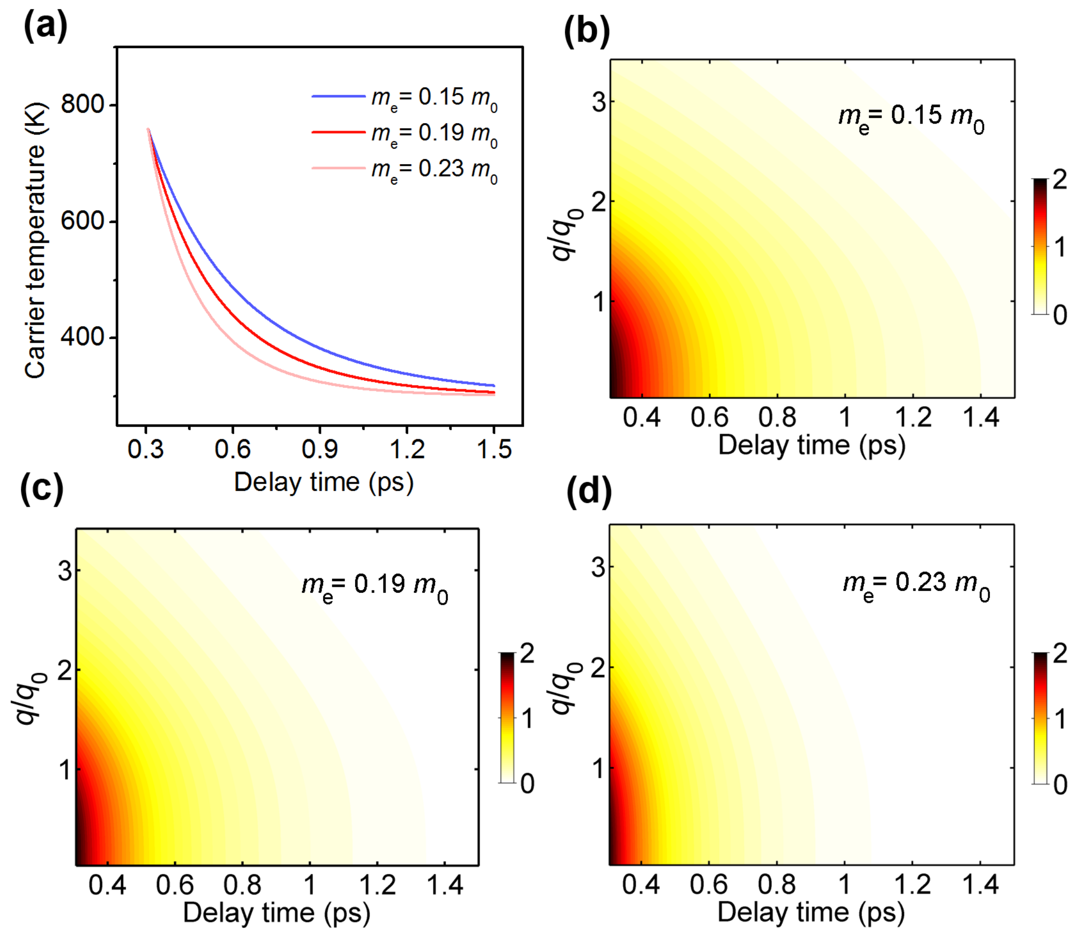

Supplementary Figure 17 Influence of electron effective mass m_e on hot carrier cooling and calculated relative non-equilibrium hot LO phonon population (*i.e.*, $\left[N_q - N_q(T_L)\right] / N_q(T_L)$)as a function of normalized phonon wave vector ($q/q0$). A lighter mass will result in a slower hot carrier cooling process. The calculations were performed for different effective electron masses (a) $m_e = 0.15\ m_0$, **(b)** $m_e = 0.19\ m_0$, and **(c)** $m_e = 0.23\ m_0$ at the same carrier concentration of 4.2×10^{18} cm^{-3}.[5-7] See Supplementary Note 8.

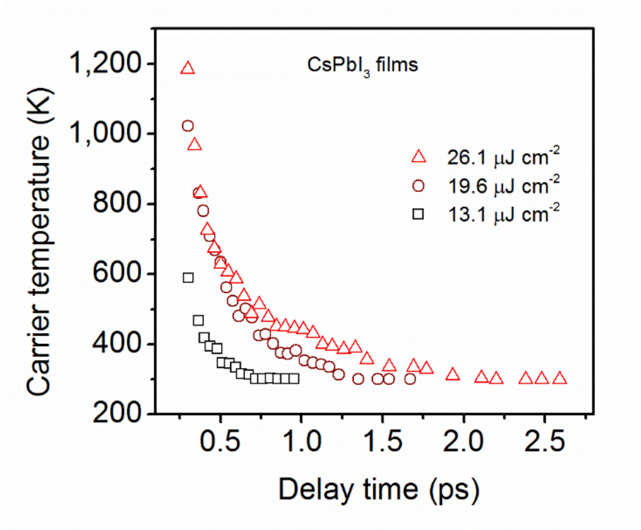

Supplementary Figure 18 HC cooling dynamics of $CsPbI_3$ films excited at 2.48 eV with different pump fluences. At high pump fluence, the slope of carrier cooling becomes gentler as hot carriers take more time to cool down to the lattice. This indicates the presence of hot phonon effect. Such similar observations as that for $MAPbI_3$ suggests that the organic cation may not play a significant role in the slow hot carrier cooling.

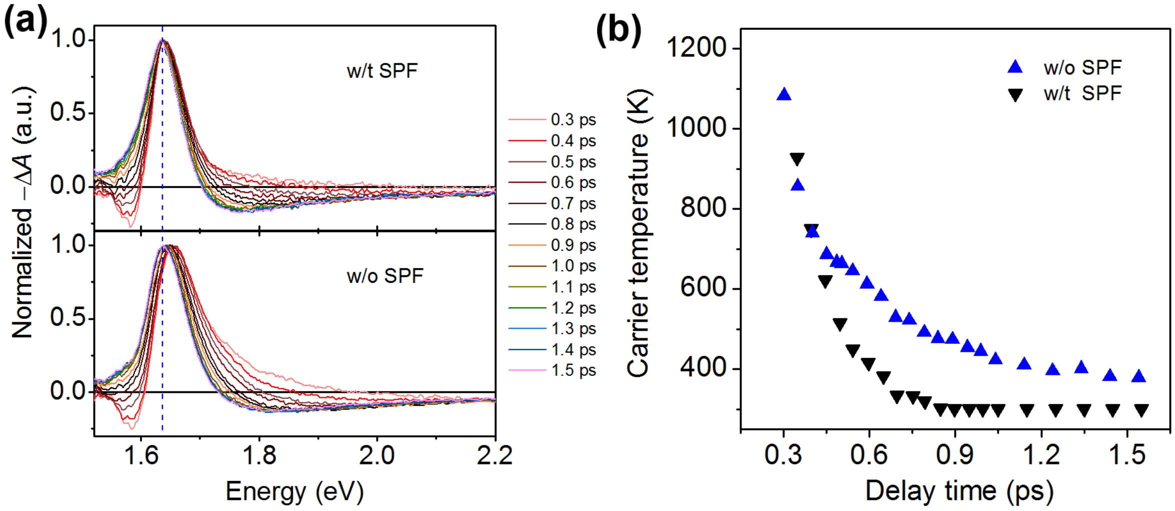

Supplementary Figure 19 (a) Influence of 750 nm short pass filter (SPF) on TA spectra (top (with SPF filter) and bottom (without SPF filter)) and **(b)** HC cooling dynamics with pump energy of 2.48 eV and carrier concentration n_0 of 1.6×10^{18} cm^{-3}.

Supplementary Table 1: List of parameters relating to the hot-phonon effect for different semiconductors. The studies were performed at room temperature unless stated otherwise in parenthesis in column 1.

Semiconductor	LO Phonon lifetime (ps)	Hot carrier cooling lifetime (ps)	Carrier density (cm^{-3} or cm^{-2} for 3D/2D systems) *	LO phonon energy (meV)	Methods
GaAs	~2.1	~1.85	$\sim 2\times10^{18}$	~ 36	Transient transmission[8]
GaAs quantum wells	—	~ 3	$\sim 1\times10^{12}$	~ 36	Fluorescence up-conversion[9]
InP	7.6	~ 1.5	$\sim 5\times10^{17}$	~ 43	Transient absorption & Time-resolved luminescence[10]
ZnO	~ 1.75	0.4 ~ 1.0	$> 8\times10^{18}$	~ 70	Transient absorption[11]
GaN	0.5 ~ 2.5	~ 0.6	$\sim 10^{18}$	92	Time-resolved Raman[12]
$Al_xGa_{1-x}N$/GaN multiple wells	0.35	—	$\sim 5\times10^{12}$	~ 92	Short-time-domain gated radiometric microwave noise technique[13]
CdSe (8 K)	~ 5	~ 10	$\sim 2\times10^{17}$	~ 26.5	Fluorescence up-conversion (~2.5 ps)[14]
CdS	~ 0.6	~ 2	$\sim 3\times10^{17}$	~ 35	Fluorescence up-conversion[15]
$MAPbBr_3$ nanocrystals	—	0.5 ~ 1.0	Less than $\langle N_0 \rangle \sim 0.1$ or $n_{0avg} \sim 2.6\times10^{17}$	~ 15.3 [16]	Transient absorption [17]
$MAPbI_3$ thin films	~ 0.6	0.3 ~ 1.0	$\sim 6\times10^{17}$	~ 13	Transient absorption (This work)

* Carrier density when hot phonon effect is observed

As discussed in our earlier work[17], the measured hot-phonon lifetime could also be limited by the time-resolution of the experimental techniques used, thereby yielding artificially longer lifetimes that are limited by the system temporal response rather than its intrinsic hot-phonon lifetime. Hence, due care must be taken for a fair comparison of the reported values in the literature.

Supplementary Notes

Supplementary Note 1. *Analysis of absorption spectra*

The absorption coefficient of the sample is calculated using the following equation[18,19]:

$$\alpha_{\mathrm{film}} = \frac{d_{\mathrm{sub}}}{d_{\mathrm{tot}}} \frac{1}{d_{\mathrm{film}}} \ln\left(\frac{1-R_{\mathrm{tot}}}{T_{\mathrm{tot}}}\right) - \frac{1}{d_{\mathrm{sub}}} \ln\left(\frac{1-R_{\mathrm{sub}}}{T_{\mathrm{sub}}}\right) \tag{1}$$

where R_{tot}, T_{tot} and d_{tot} are the reflectance, transmittance and thickness of the film/substrate structure, respectively. R_{sub}, T_{sub} and d_{sub} are reflectance, transmittance and thickness of quartz substrates, d_{film} is the thickness of perovskite films.

The band edge absorption spectrum consisting of continuum and discrete transition is fitted using the Elliott formula[20].

$$\alpha(E) = \frac{(2\mu)^{3/2} e^2 f_{\mathrm{cv}}}{\tilde{n} c \hbar^2 m_0} \left[\sum_n 4\pi R_0^{*3/2}\, \delta(E - E_n) / n^3 + \frac{2\pi R_0^{*1/2} \theta(E - E_n)}{1 - \exp(-2\pi \sqrt{\frac{R_0^*}{E - E_{\mathrm{g}}}})} \right] \tag{2}$$

where E_{g} is the bandgap, μ is the reduced mass, m_0 is the free electron mass, $\tilde{n}$ is the refractive index, f_{cv} is the oscillator strength, $\theta(x)$ is the unit step function, R_0^* is the effective Rydberg. The estimated bandgap and the exciton binding energy are respectively 1.651±0.001 eV and 6.3±0.1 meV, which is in agreement with the measured value using high magnetic fields[21]. The negligible binding energy as well as insignificant exciton contribution to the band edge absorption indicates a Wannier-type exciton in $MAPbI_3$ films and immediate dissociation of

exciton into free carriers after photoexcitation, which to some degree explains the excellent performance of lead iodide perovskite $MAPbI_3$ in photovoltaic applications.

Supplementary Note 2. *Estimation of trap density*

The carrier density is estimated by $n_0 = \frac{P\alpha}{\hbar\omega}$, where P is the pump fluence, α is the thin film absorption coefficient, $\hbar\omega$ is the photon energy. Time integrated PL were measured at relative low pump fluence in order to minimize the Auger effects. The trap density was estimated from the following equation[22]:

$$n_0 = \sum_i n_{\mathrm{TP}}^i(0)\left(1 - e^{-a_i \tau_0 I_{\mathrm{PL}}/\mathrm{k}}\right) + I_{\mathrm{PL}}/k \quad (3)$$

where $n_{\mathrm{TP}}^i(0)$ is the initial trap states density, a_i is the product of the trapping cross section and the carrier velocity, I_{PL} is the integrated PL intensity, k is a constant. The estimated trap density of the perovskite film is $n_{\mathrm{TP}}^i(0) = 8.9 \pm 0.6 \times 10^{16}\ \mathrm{cm}^{-3}$, which is exceptionally low for solution prepared films - indicating the excellent properties of $MAPbI_3$ for optoelectronic applications.

Supplementary Note 3. *Model for band gap shifting*

Generally, there are four factors contributing to the band gap shift in a semiconductor: band gap renormalization (BGR) originating from strong electron or hole exchange interactions; decreasing of exciton binding energy, temperature sensitive lattice expansion or contraction and Burstein-Moss (MB) effects stemming from Pauli exclusion principle[23]. Given the short laser pulse used here, we do not expect considerable rise in lattice temperature by laser heating. The

carrier density dependent BGR which results in shrinkage of bandgap can be described by $\Delta E_{\mathrm{BGR}} = \Delta E_{\mathrm{ee}} + \Delta E_{\mathrm{ei}}$, where the contributions from electron-electron interactions and electron-ion interactions are respectively $\Delta E_{\mathrm{ee}} = -\frac{e^2 k_{\mathrm{F}}}{2\pi^2 \varepsilon_0 \varepsilon_{\mathrm{s}}} - \frac{e^2 k_{\mathrm{F}}}{8\pi^2 \varepsilon_0 \varepsilon_{\mathrm{s}}}\left[1 - \frac{4}{\pi}\arctan\left(\frac{k_{\mathrm{F}}}{k_{\mathrm{TF}}}\right)\right]$ and $\Delta E_{\mathrm{ei}} = -\frac{e^2 n}{\varepsilon_0 \varepsilon_{\mathrm{s}} a^*_{\mathrm{B}\text{-}} k^3_{\mathrm{TF}}}$[23]. Here, $k_{\mathrm{F}} = (3\pi^2 n)^{1/3}$ is the momentum at Fermi surface, ε_0 and ε_{s} are respectively the vacuum dielectric constant and static dielectric constant of the perovskite, $k_{\mathrm{TF}} = 2\sqrt{k_{\mathrm{F}}/\pi a^*_{\mathrm{B}\text{-}}}$ is the estimated inverse Thomas-Fermi screening length, $a^*_{\mathrm{B}\text{-}} = \frac{4\pi\varepsilon_0\varepsilon_{\mathrm{s}}\hbar^2}{m^*_{\mathrm{DE}} e^2}$ is the screened Bohr radius, m^*_{DE} is the degenerate effective mass. Considering the carrier density of interest investigated here, we do not expect the screening effect to play a significant role. Assuming a parabolic band structure, the blue shift of bandgap due to Burstein-Moss effects for the degenerate electron-hole gas $\Delta E_{\mathrm{BMS}} = \frac{\hbar^2}{2\mu} k^2_{\mathrm{F}}$,[23] where $\hbar$ is the reduced Planck's constant, $\mu = \left(\frac{1}{m_{\mathrm{e}}} + \frac{1}{m_{\mathrm{h}}}\right)^{-1}$ is the reduced mass, and n is the carrier density. The contribution from the reduction of free exciton binding energy can be described by: $E_{\mathrm{bX}} = \frac{e^2}{8\pi\varepsilon_0\varepsilon_{\mathrm{S}} a^*_{\mathrm{B}}} - \frac{3e^2(3\pi^2)^{1/3}}{(4\pi)^2 \varepsilon_0 \varepsilon_{\mathrm{S}}} n^{1/3}$. The band gap of the semiconductor after photoexcitation with all the contributions taken into account is: $E(n) = E_{\mathrm{gap}} - E_{\mathrm{bX}} + \Delta E_{\mathrm{BGR}} + \Delta E_{\mathrm{BMS}}$.

Supplementary Note 4. *Analysis of TA spectra and hot carrier distribution*

From Fermi's golden rule, the absorption coefficient for a direct semiconductor within a parabolic band approximation is:

$$\alpha(E, E_{\mathrm{g}}) = C_{\mathrm{cv}} \frac{\rho_{\mathrm{cv}}(E, E_{\mathrm{g}})}{E} [f_{\mathrm{v}}(E_{\mathrm{v}}) - f_{\mathrm{c}}(E_{\mathrm{c}})] \tag{4}$$

where C_{cv} is a constant related with transition matrix, $\rho_{cv}\left(E,E_g\right)=\frac{1}{2\pi^2}\left(\frac{2m_r}{h^2}\right)^{3/2}\sqrt{E-E_g}$ is the joint density of states with the band gap of E_g, $f_{c,v}\left(E_{c,v}\right)=\left[1+\exp[(E_{c,v}-E_{Fc,v})/(k_B T)]\right]^{-1}$ is the Fermi-Dirac distribution function of the electron in the conduction band and valence band, m_r is the reduced mass, h is the Plank constant, k_B is the Boltzmann constant, $E_{Fc,v}$ is the quasi-Fermi energy of the electron. Populating the semiconductor by photoexcitation or injection will modify the absorption coefficient by changing the Fermi distribution of the carriers via bandgap renormalization and band filling effects[24]. However, at the short timescale (< 2 ps), the tradeoff from bandgap renormalization (causing red-shift) and Burstein-Moss effects (causing blue-shift) result in a negligible shift of the bandgap (Supplementary Figure 6), the joint density states $\rho_{cv}\left(E,E_g\right)$ are therefore rarely affected by the band gap variation. ΔA can then be approximated by:[25]

$$\Delta A(E)=-A_0(E)\left[f_e(E_c)+f_h(E_v)\right] \tag{5}$$

where $A_0(E)$ is steady state linear absorbance. One therefore will always obtain a negative ΔA contributed by both the electrons and the holes. Meanwhile, from Supplementary Equation 5 we can see that the profile of ΔA correlates straightforwardly with Fermi distributions of the electrons and the holes. Given their similar effective masses[7] ($m_e = 0.19\,m_0, m_h = 0.25\,m_0$), their contributions to the hot carrier cooling process will be roughly the same (*i.e.*, $f_e \approx f_h$). Inclusion of this difference only slightly changes the prefactor of 2 for the exponential in Supplementary Equation 5. Most importantly, this will not affect the derived hot carrier temperature (that is embedded within the exponential term). Hence, for convenience, we assume that the extracted

HC temperature to be the electron's ($T_c \approx T_e$), which will not change the physics and the conclusions here.

Supplementary Note 5. *Determination of first order, second order, and third order recombination coefficients*

TRPL measurement was conducted at relative low fluence to minimize any Auger effects on the charge carrier dynamics. The carrier dynamics after photoexcitation can then be expressed by:

$$\frac{dn}{dt} = -k_1 n - k_2 n^2 \tag{6}$$

where k_1 is the monomolecular recombination coefficient which correlates with free carrier trapping, k_2 is the bimolecular recombination coefficient which is related with photoluminescence. From the instantaneous PL intensity $I_{\mathrm{PL}} = k_2 n^2$, the carrier lifetime can therefore be obtained[26]:

$$\frac{1}{\tau_{\mathrm{PL}}} = \left|\frac{d\log(I_{\mathrm{PL}})}{dt}\right|_{t=0} = 2(k_2 n_0 + k_1) \tag{7}$$

The estimated monomolecular recombination coefficient is $k_1 = 2.3 \pm 0.1 \times 10^7$ s^{-1}. The Auger recombination coefficient corresponding to three body interactions is estimated from carrier concentration dependent band edge photobleaching ΔA kinetics. $\Delta A \propto n$ since ΔA is dominated by band filling effects within the appropriate excitation range. The charge carrier dynamics can be described by:

$$\frac{dn}{dt} = -k_1 n - k_2 n^2 - k_3 n^3 \tag{8}$$

where k_3 is the Auger coefficient. We global fit the ΔA kinetics obtained at different carrier densities. The monomolecular recombination rate k_1 was obtained from TRPL measurement (Supplementary Figure 12 (c)). The estimated bimolecular recombination coefficient and Auger coefficient are respectively $k_2 = 2.5 \pm 0.2 \times 10^{-11}\ \mathrm{cm^3\ s^{-1}}$ and $k_3 = 5.4 \pm 0.1 \times 10^{-29}\ \mathrm{cm^6\ s^{-1}}$, which are in agreement with literature values.[27,28]

Supplementary Note 6. *Model for Coulomb screening effect*

We utilized a static random phase approximation (RPA) method to examine the influence of screening effect on HC cooling in the presence of the hot phonon bottleneck. The screened electron-phonon matrix element can be described by[29,30]:

$$\left|M_q\right|^2 = \frac{2\pi \hbar^2 e E_0}{m_e q^2 \left(1 + q_{\mathrm{RPA}}^2 / q^2\right)^2} \tag{9}$$

where the inverse squared screening length $q_{\mathrm{RPA}}^2 = \frac{ne^2}{\varepsilon_0 \varepsilon_{\mathrm{Stat}} k_B T_c} \sum_i \frac{F_{-1/2}(\eta)}{F_{1/2}(\eta)}$, $F_j(x)$ is Fermi integral of the jth order which is given by $F_j(\eta) = \frac{1}{\Gamma(j+1)} \int_0^\infty \frac{\xi^j d\xi}{1 + e^{\xi - \eta}}$, $\Gamma(j+1)$ is the jth order gamma function. We calculated the screened HC cooling dynamics using the modified matrix element at the highest excitation densities for our experiments (Supplementary Figure 13). Compared to the hot phonon effect, influence of free carrier screening effect has a negligible effect on HC cooling. Therefore, we conclude here that slow HC cooling in halide perovskites is largely unaffected by free carrier Coulomb screening effect over the carrier densities of $10^{17} - 10^{19}\ \mathrm{cm^{-3}}$.

Supplementary Note 7. *Estimation of electron-LO phonon scattering time constant*

The electron-LO phonon scattering time constant is estimated from the following equation[31]:

$$\tau^{-1} = \frac{e^2\sqrt{2m\hbar\omega_{\mathrm{LO}}}}{4\varepsilon_0\pi\hbar^2}\left(\frac{1}{\varepsilon_\infty} - \frac{1}{\varepsilon_{\mathrm{S}}}\right) \tag{10}$$

where m is the effective mass of the electron, $\hbar\omega_{\mathrm{LO}}$ is the LO phonon energy, ε_0 is the vacuum permittivity, ε_∞ and ε_{S} are respectively the high frequency and static dielectric constants. The scattering time constant is estimated to be around 13 fs.

Supplementary Note 8. *Dependence on the electron effective mass*

As for the dependence of hot carrier cooling on the effective electron mass, the scenario can be understood from the energy loss of the hot carriers in the absence of hot phonon effect. The energy loss rate of the hot carriers can be expressed by:

$$P_0 = eE_0\left(\frac{2\hbar\omega_{\mathrm{LO}}}{m}\right)^{\frac{1}{2}} = \frac{m^{1/2}e^2}{\pi\varepsilon_0\hbar^2}\left(\frac{\hbar\omega_{\mathrm{LO}}}{2}\right)^{3/2}\left[\frac{1}{\varepsilon_{\mathrm{Opt}}} - \frac{1}{\varepsilon_{\mathrm{Stat}}}\right] \tag{11}$$

which means that the lighter the mass, the smaller the energy loss rate for the hot carriers. Thus, a lighter mass will lead to less efficient hot carrier relaxation and more significant hot phonon bottleneck effect. To demonstrate the influence of the effective mass on hot carrier cooling, we calculate the hot carrier cooling dynamics and the relative non-equilibrium LO phonon population for $m_{\mathrm{e}} = 0.15\ m_0$,[5] $m_{\mathrm{e}} = 0.19\ m_0$,[7] and $m_{\mathrm{e}} = 0.23\ m_0$ [6] at a carrier concentration n_0 of 4.2×10^{18} cm^{-3}. As shown in Supplementary Figure 17, a lighter mass will result in a slower hot carrier cooling process. This is consistent with the low DOS of the valence band[32] in lead iodide

perovskites $APbI_3$ (A can be CH_3NH_3, $NH_2CH{=}NH_2$ and Cs), which also leads to slow hot hole cooling.

In light of the relation between the effective mass and the hot carrier cooling rate, the similarity of perovskite's electron and hole effective masses aptly highlights its distinct advantage over conventional semiconductors for hot carrier applications. In typical semiconductors, their electron effective mass is much lower than that of the hole. This will result in faster hot hole cooling compared to hot electron cooling. In contrast, the more "balanced" slow hot electron and hot hole cooling in perovskites will be more amenable for developing practical hot-carrier optoelectronic devices.

Supplementary Note 9. *Calculated Phonon spectra and projected density of states*

Using the first-principles method based on density functional theory, we calculated the phonon dispersion spectra and the corresponding projected density of states (pDOS) of the $MAPbI_3$ in the tetragonal and orthorhombic phases, which are shown in Supplementary Figure 14. We find that both phonon spectra for the two phases are similar in low frequency (less than 16 meV). Two LO phonon modes, *i.e.*, Pb-I stretch vibrations are observed in both phases (marked as yellow zones in Supplementary Figure 14). One is located at lower frequency (around 8 meV), which are mainly dominated by Pb-I stretch vibrations. The LO phonons with higher frequency (around 12 meV) are mixed vibrations, including Pb-I stretch vibrations and organic molecule MA vibrations. We schematically show three LO phonon vibration modes of tetragonal $MAPbI_3$ in Supplementary Figure 15, where the Pb-I stretch vibrations are clearly observed. For comparison with the experiments, we also simulated the Raman spectra through computing the changes of macroscopic dielectric tensor according to each phonon mode of the system. The

results are shown in Supplementary Figure 16 (red line), which are broadened by convolving with a Lorentzian function with FWHM 8 cm^{-1}.

Supplementary References

1 Sum, T. C. *et al.* Spectral Features and Charge Dynamics of Lead Halide Perovskites: Origins and Interpretations. *Acc. Chem. Res.* **49**, 294-302 (2016).

2 Ledinský, M. *et al.* Raman spectroscopy of organic–inorganic halide perovskites. *J. Phys. Chem. Lett.* **6**, 401-406 (2015).

3 Leguy, A. M. A. *et al.* Dynamic disorder, phonon lifetimes, and the assignment of modes to the vibrational spectra of methylammonium lead halide perovskites. *Phys. Chem. Chem. Phys.* **18**, 27051-27066 (2016).

4 Comin, R. *et al.* Lattice dynamics and the nature of structural transitions in organolead halide perovskites. *Phys. Rev. B* **94**, 094301 (2016).

5 Mosconi, E., Umari, P. & De Angelis, F. Electronic and optical properties of mixed Sn–Pb organohalide perovskites: a first principles investigation. *J. Mater. Chem. A* **3**, 9208-9215 (2015).

6 Giorgi, G., Fujisawa, J.-I., Segawa, H. & Yamashita, K. Small photocarrier effective masses featuring ambipolar transport in methylammonium lead iodide perovskite: a density functional analysis. *J. Phys. Chem. Lett.* **4**, 4213-4216 (2013).

7 Umari, P., Mosconi, E. & De Angelis, F. Relativistic GW calculations on $CH_3NH_3PbI_3$ and $CH_3NH_3SnI_3$ perovskites for solar cell applications. *Sci. Rep.* **4**, 4467 (2014).

8 Langot, P., Del Fatti, N., Christofilos, D., Tommasi, R. & Vallée, F. Femtosecond investigation of the hot-phonon effect in GaAs at room temperature. *Phys. Rev. B* **54**, 14487 (1996).

9 Pelouch, W. *et al.* Comparison of hot-carrier relaxation in quantum wells and bulk GaAs at high carrier densities. *Phys. Rev. B* **45**, 1450 (1992).

10 Hohenester, U. *et al.* Subpicosecond thermalization and relaxation of highly photoexcited electrons and holes in intrinsic and p-type GaAs and InP. *Phys. Rev. B* **47**, 13233 (1993).

11 Sun, C.-K. *et al.* Ultrafast carrier dynamics in ZnO nanorods. *Appl. Phys. Lett.* **87**, 023106 (2005).

12 Ye, H., Wicks, G. & Fauchet, P. Hot electron relaxation time in GaN. *Appl. Phys. Lett.* **74**, 711-713 (1999).

13 Matulionis, A. *et al.* Hot-phonon temperature and lifetime in a biased $Al_xGa_{1-x}N$/GaN channel estimated from noise analysis. *Phys. Rev. B* **68**, 035338 (2003).

14 Prabhu, S., Vengurlekar, A., Roy, S. & Shah, J. Nonequilibrium dynamics of hot carriers and hot phonons in CdSe and GaAs. *Phys. Rev. B* **51**, 14233 (1995).

15 Klimov, V., Bolivar, P. H. & Kurz, H. Hot-phonon effects in femtosecond luminescence spectra of electron-hole plasmas in CdS. *Phys. Rev. B* **52**, 4728 (1995).

16 Wright, A. D. *et al.* Electron-phonon coupling in hybrid lead halide perovskites. *Nat. Commun.* **7**, 11755 (2016).

17 Li, M. *et al.* Slow cooling and highly efficient extraction of hot carriers in colloidal perovskite nanocrystals. *Nat. Commun.* **8**, 14350 (2017).

18 Cesaria, M., Caricato, A. & Martino, M. Realistic absorption coefficient of ultrathin films. *J. Opt.* **14**, 105701 (2012).

19 Xing, G. *et al.* Long-range balanced electron-and hole-transport lengths in organic-inorganic $CH_3NH_3PbI_3$. *Science* **342**, 344-347 (2013).
20 Sell, D. D. & Lawaetz, P. New Analysis of Direct Exciton Transitions: Application to GaP. *Phys. Rev. Lett.* **26**, 311-314 (1971).
21 Miyata, A. *et al.* Direct measurement of the exciton binding energy and effective masses for charge carriers in organic-inorganic tri-halide perovskites. *Nature Phys.* **11**, 582-587 (2015).
22 Xing, G. *et al.* Low-temperature solution-processed wavelength-tunable perovskites for lasing. *Nat. Mater.* **13**, 476-480 (2014).
23 Feneberg, M. *et al.* Band gap renormalization and Burstein-Moss effect in silicon-and germanium-doped wurtzite GaN up to 10^{20} cm^{-3}. *Phys. Rev. B* **90**, 075203 (2014).
24 Price, M. B. *et al.* Hot-carrier cooling and photoinduced refractive index changes in organic-inorganic lead halide perovskites. *Nat. Commun.* **6**, 8420 (2015).
25 Bennett, B. R., Soref, R. A. & Del Alamo, J. A. Carrier-induced change in refractive index of InP, GaAs and InGaAsP. *IEEE J. Quant. Electron.* **26**, 113-122 (1990).
26 Jordan, C. *et al.* Carrier-density dependence of the photoluminescence lifetimes in ZnCdSe/ZnSSe quantum wells at room temperature. *Appl. Phys. Lett.* **74**, 3359-3361 (1999).
27 Milot, R. L., Eperon, G. E., Snaith, H. J., Johnston, M. B. & Herz, L. M. Temperature‐Dependent Charge‐Carrier Dynamics in $CH_3NH_3PbI_3$ Perovskite Thin Films. *Adv. Funct. Mater.* **25**, 6218-6227 (2015).
28 Johnston, M. B. & Herz, L. M. Hybrid perovskites for photovoltaics: Charge-carrier recombination, diffusion, and radiative efficiencies. *Acc. Chem. Res.* **49**, 146-154 (2015).
29 Pugnet, M., Collet, J. & Cornet, A. Cooling of hot electron-hole plasma in the presence of screened electron-phonon interactions. *Solid State Commun.* **38**, 531-536 (1981).
30 Žukauskas, A. Second nonequilibrium-phonon bottleneck for carrier cooling in highly excited polar semiconductors. *Phys. Rev. B* **57**, 15337 (1998).
31 Ridley, B. Hot phonons in high-field transport. *Semicond. Sci. Technol.* **4**, 1142 (1989).
32 Kawai, H., Giorgi, G., Marini, A. & Yamashita, K. The Mechanism of Slow Hot-Hole Cooling in Lead-Iodide Perovskite: First-Principles Calculation on Carrier Lifetime from Electron–Phonon Interaction. *Nano Lett.* **15**, 3103-3108 (2015).
